# Detection of Ammonia-Oxidizing Bacteria (AOB) Using a Porous Silicon Optical Biosensor Based on a Multilayered Double Bragg Mirror Structure

**DOI:** 10.3390/s18010105

**Published:** 2018-01-01

**Authors:** Hongyan Zhang, Jie Lv, Zhenhong Jia

**Affiliations:** 1 School of Physical Science and Technology, Xinjiang University, Urumqi 830046, China; zhanghongyanxj@163.com; 2 College of Resource and Environment Science, Xinjiang University, Urumqi 830046, China; lvjie@xju.edu.cn; 3 College of Information Science and Engineering, Xinjiang University, Urumqi 830046, China

**Keywords:** label-free DNA biosensor, porous silicon, photonic crystal, double Bragg mirror structure

## Abstract

We successfully demonstrate a porous silicon (PS) double Bragg mirror by electrochemical etching at room temperature as a deoxyribonucleic acid (DNA) label-free biosensor for detecting ammonia-oxidizing bacteria (AOB). Compared to various other one-dimension photonic crystal configurations of PS, the double Bragg mirror structure is quite easy to prepare and exhibits interesting optical properties. The width of high reflectivity stop band of the PS double Bragg mirror is about 761 nm with a sharp and deep resonance peak at 1328 nm in the reflectance spectrum, which gives a high sensitivity and distinguishability for sensing performance. The detection sensitivity of such a double Bragg mirror structure is illustrated through the investigation of AOB DNA hybridization in the PS pores. The redshifts of the reflectance spectra show a good linear relationship with both complete complementary and partial complementary DNA. The lowest detection limit for complete complementary DNA is 27.1 nM and the detection limit of the biosensor for partial complementary DNA is 35.0 nM, which provides the feasibility and effectiveness for the detection of AOB in a real environment. The PS double Bragg mirror structure is attractive for widespread biosensing applications and provides great potential for the development of optical applications.

## 1. Introduction

Nitrification, the biological oxidation of ammonium to nitrite and nitrate, is an essential process and a critical step in nitrogen cycling and different wastewater treatment bioreactors, which is mediated by a series of phylogenetically- and physiologically-distinct microorganisms [1,2,3]. Ammonia-oxidizing bacteria (AOB) are responsible for nitrification in terrestrial ecosystems, and they play important roles in ecosystem functioning by modulating the rates of nitrogen losses to ground water and the atmosphere. Although some heterotrophic bacteria and anaerobic ammonia-oxidizing bacteria can also oxidize ammonia to nitrite, AOB are thought to be the main contributors for environmental ammonia oxidation and in bioreactors [4,5,6]. The conventional methods for quantitative determining AOB DNA sequence are sensitive, but require labeling and they are time-consuming and suppress specific hybridization [2,4,5,6]. Therefore, rapid and reliable detection and identification of AOB is critical for environmental protection. 

Nowadays, research studies are continuously increasing to find new methods and devices that would provide easy, reproducible, and sensitive sensing assays for biomolecular detection. As one kind of promising candidate for label-free biosensors, compared to other sensors, like plasmonics, colorimetric chemical assays and fluorescence sensing, porous silicon (PS) is attracting many researchers due to its large internal surface area, tunable optical properties, good mechanical robustness, and compatibility with integrated circuit (IC) technology [7,8,9]. Many researchers have investigated various aspects of PS, such as morphological, optical, and electrical properties, especially in structural properties based on the preparation of integrated optical devices [8,9,10]. Currently, photonic crystal structures are widely used as transducers for optical sensing applications based on controlling the refractive index of the PS layers and the change in the effective refractive index due to biomolecule attachment events [11,12]. The dielectric functions of periodically-modulated photonic crystals results in light propagation dramatically different from the bulk materials, such as waveguides, Bragg mirrors, rugate filters, and Fabry-Perot filters [13,14]. Many groups have performed research on sensors with different photonic crystal structures to obtain the highest sensitivity [15,16,17]. However, the development of sensors using PS is limited by several technical problems, and it is difficult to obtain the proper results consistent with simulations. One of the main problems is that there are many external random fluctuations produced in the electrochemical etching process in the fabrication of PS photonic crystals, where some unpredicted surface irregularities and impurities will affect the thickness and complex refractive index of each PS layer. Any loss presented inside a fabricated PS photonic crystal due to optical absorption, interface scattering between adjacent PS layers, and bulk scattering from within layers may alter the cavity spectral properties. Furthermore, there are transverse chemical etching and axial electrochemical etching in the process of electrochemical etching. The transverse chemical etching will destroy the homogeneity of the PS photonic crystal more during the PS layer formation when the process goes deeper [18,19]. Though the difference of the variation for both axial and transverse etching between alternative layers is not evident at first, with the increase of layers, the accumulated change will be large enough to affect the characteristics and spectral properties of PS photonic crystal. According to the literature, many researchers have obtained high-quality PS photonic crystals through specific preparation conditions, such as low temperature anodic oxidation [20]. Hence, from both theoretical and practical considerations, studying a simple and controllable new structure of PS photonic crystals is very important for a wide range of applications.

In this paper, we demonstrate a simple and controllable PS double Bragg mirror structure following the (n_L1_n_H1_)^9^(n_L2_n_H2_)^9^ sequence by electrochemical etching at room temperature with high sensitivity for AOB DNA detection. Various numerical analysis has been achieved to investigate the effect of any change in the thickness and refractive index of different layers on the spectral properties of the fabricated double Bragg mirror before starting detection and identification applications. From experimental results, there is a deeper resonance peak and a broader reflectivity stop band in the reflectance spectrum of the double Bragg mirror structure compared to other PS photonic crystal structures, which is more suitable for evident variations of optical properties when exposed to target biomolecules and to achieve highly-sensitive biosensors. Moreover, considering the importance of transmission at a 1310 nm wavelength for the telecommunication industry, and the very mature preparation technology for optical devices working at this wavelength [21,22,23], we chose the optical wavelength as 1310 nm with a narrow resonance peak for the double Bragg structure. Therefore, the photonic crystal with such a structure provides an interesting method to develop a new optical label-free biosensor, which has great potential for a wide range of optical applications.

## 2. Materials and Methods

### 2.1. Materials and Instruments

Four-hundred micrometer thick p-type (1 0 0) silicon with a resistivity of 0.03–0.06 Ω·cm was purchased from Tianjin Institute of Semiconductors (Tianjing, China). 3-aminopropyltriethoxyailane (APTES), 50% glutaraldehyde, and ethanolamine were purchased from Aladdin Reagent Co., Shanghai, China. All the chemical reagents were of analytical grade and used without further purification. Deionized water was used throughout the fabrication of the PS double Bragg mirror.

**Figure 1:**
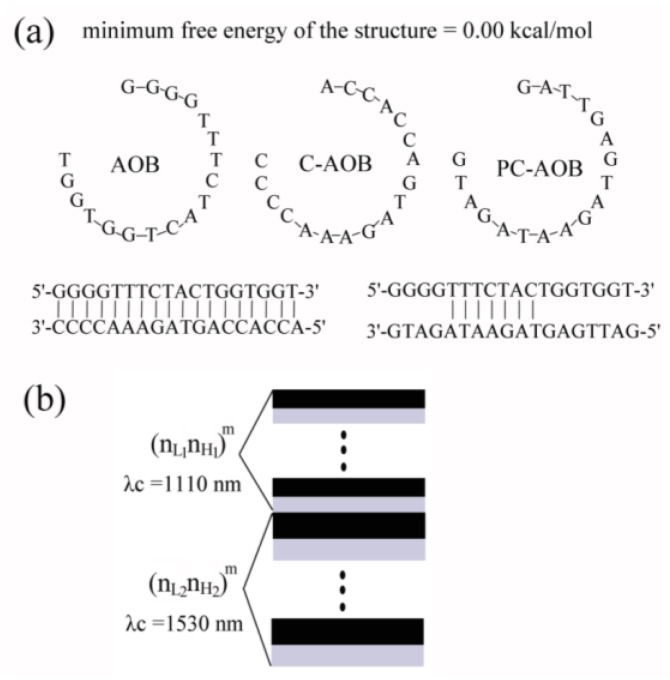
(**a**) Scheme of the sequences and the structure of AOB DNA. (**b**) Scheme and parameters of the double Bragg mirror structure.

To ensure the specificity and accuracy of the biosensor, we detected three target DNA with different sequences: one is a sequence of DNA that is completely complementary to the probe DNA, one is a sequence of DNA that has only seven base pairs in the middle complementary to the probe DNA, and the other is of completely non-complementary DNA. AOB DNA oligonucleotides were purchased from Invitrogen (Shanghai, China). The oligonucleotide DNA sequences of AOB were as follows, and the structure is shown in Figure 1. The sequence of the probe DNA (AOB, amoA-1F), i.e., amino-modified bacterial AOB gene, is (5′-GGGGTTTCTACTGGTGGT-(CH_2_)_3_-NH_3_-3′) (18 based). There is good amplification and there is no complementarity in the primer itself. The partial complementary DNA sequence (PC-AOB) is 5′-GATTGAGTAGAATAGATG-3′ (18 based), and there is no complementarity in the primer itself. The complementary DNA sequence (C-AOB) is 5′-ACCACCAGTAGAAACCCC-3′ (18 bp), and the non-complementary DNA (N-AOB) is 5′-ATTTGAACTGGTGACACGAG-3′ (20 bp). All oligonucleotides were purchased from Invitrogen Trading Co., Ltd. (Shanghai, China). Reflectance spectra were measured by a U-4100 UV-VIS scanning spectrophotometer (Hitachi, Tokyo, Japan) with the wavelength ranging from 240.00 to 2600.00 nm, and the incident angle is 5 ± 1°. The illumination with a focused beam size is approximately 2.2 (W) × 2.2 (H) mm and the sample mounting section is 20 mm. Field emission scanning electron microscope (FESEM) images were obtained from an S-4800 scanning electron microscope (Hitachi, Japan). 

### 2.2. Simulations

**Figure 2:**
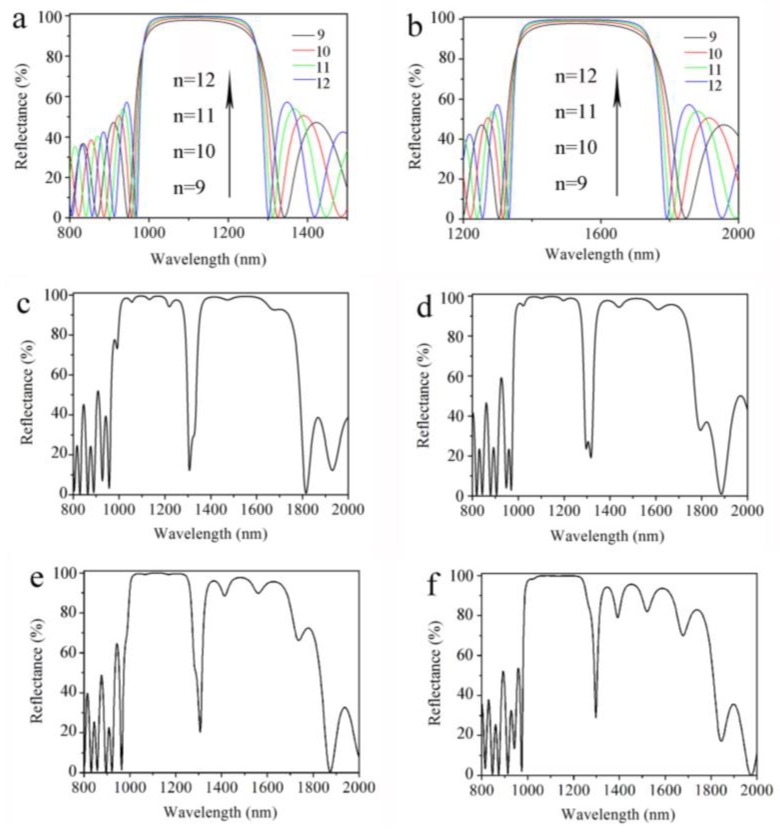
Calculated reflectance spectra of the Bragg mirror structure following the (n_L_n_H_)^n^ sequence (n is the number of periods) with the resonance wavelength at (**a**) 1310 nm and (**b**) 1530 nm, respectively. Calculated reflectance spectra of the double Bragg mirror structure following (**c**) (n_L1_n_H1_)^9^(n_L2_n_H2_)^9^; (**d**) (n_L1_n_H1_)^10^(n_L2_n_H2_)^10^; (**e**) (n_L1_n_H1_)^11^(n_L2_n_H2_)^11^; and (**f**) (n_L1_n_H1_)^12^(n_L2_n_H2_)^12^ sequences, with the resonance wavelength of (n_L1_n_H1_)^m^ at 1310 nm and the resonance wavelength of (n_L2_n_H2_)^m^ at 1530 nm, respectively.

Figure 1b shows a schematic cross-section of the proposed double Bragg mirror structure following the (n_L1_n_H1_)^m^(n_L2_n_H2_)^m^ sequence, with n_L1_ and n_L2_ denoting the low refractive index layers, n_H1_ and n_H2_ representing the high refractive index layers, respectively, and m being the number of periods. In order to study and obtain the high-quality PS double mirror structure with high sensitivity, simulations of the reflectance spectrum of the Bragg mirror and the double Bragg mirror had been calculated, as shown in Figure 2, by the transfer matrix method. The resolution of the grid in the calculated reflectance spectrum was set to be 1 nm. Following our earlier work, the refractive indices were determined by experiment to be n_L1_ = n_L2_ = 1.4 and n_H1_ = n_H2_ = 2.0, respectively, and the layer thickness related to the reflective indexes were d = λc/(4n) by Bruggemann’s effective medium theory [11,14,17].

Figure 2a,b, we used transfer matrix method by Matlab software to calculate reflectance spectra of the Bragg mirror of (n_L_n_H_)^9^, (n_L_n_H_)^10^, (n_L_n_H_)^11^, and (n_L_n_H_)^12^ sequences with resonance wavelengths at 1110 nm and 1530 nm, respectively. The transfer matrix method can be found in more detail in [11,14,17]. We can observe a high reflectivity stop at 1110 nm and 1530 nm with interference fringes in the reflectance spectrum. With the increase of the period from nine to 12, the reflectivity of the Bragg reflector increased from 97.8% to 99.8%, the width of the reflectivity stop band decreased, and the boundary of the reflectance spectrum became more vertical. As a comparison, the reflectance spectra of the double Bragg mirror following the (n_L1_n_H1_)^9^(n_L2_n_H2_)^9^, (n_L1_n_H1_)^10^(n_L2_n_H2_)^10^, (n_L1_n_H1_)^11^(n_L2_n_H2_)^11^, and (n_L1_n_H1_)^12^(n_L2_n_H2_)^12^ sequences are calculated in Figure 2c–f. From the simulations, the resonance wavelength of (n_L1_n_H1_)^m^ is at 1310 nm and the resonance wavelength of (n_L2_n_H2_)^m^ is at 1530 nm. Compared to other double Bragg mirror structures, the optical characteristics of the (n_L1_n_H1_)^9^(n_L2_n_H2_)^9^ sequence shows a higher and broader reflectivity stop band with one narrow resonance peak of transmittance approximately in its center. At the same time, the number of periods also affects the optical characteristics and resonance defect of double Bragg mirrors. The resonance dip becomes sharper and the reflectance of the reflectivity stop band increases gradually with the increase in the number of periods. This comes from the slight difference of the reflectance boundary of the Bragg mirror with different numbers of periods. It is important that the position and the characteristics of the narrow peak are determined by resonance wavelengths of the two Bragg mirrors of (n_L1_n_H1_)^m^ and (n_L2_n_H2_)^m^. From our simulation, adjusting the resonance wavelength of two Bragg mirrors will cause a resonance dip in the reflectance spectrum. It can be seen from the calculated reflectance spectra of (n_L1_n_H1_)^9^(n_L2_n_H2_)^9^ sequence in Figure 2c, the width of the high-reflectivity stop band is about 800 nm and the minimum reflectivity of the resonance dip is about 20%. 

### 2.3. Fabrication

The fabrication of one-dimensional photonic crystal of PS can be referenced from our early works [11,14,17]. In this experiment, PS layers have been produced with highly p-type silicon (*ρ* = 0.01–0.06 Ω·cm) with an exposing area of approximately 0.785 cm^2^ by electrochemical etching at room temperature (RT). The electrochemical etching solution is composed of 49% aqueous hydrofluoric acid and ethanol (1:1, in volume). The double Bragg mirror structure was obtained by the (n_L1_n_H1_)^m^(n_L2_n_H2_)^m^ sequence, where the number of periods m was 9, 10, 11, and 12, respectively. The different refractive index layers were fabricated by using a computer program Labview to alternately change the anodization current for different times. The n_L1_ layer was formed at a current density of 80 mA/cm^2^ for 3 s, 20 mA/cm^2^ of 4.1 s for the n_H1_ layer, 80 mA/cm^2^ of 4.1 s for the n_L2_ layer, 20 mA/cm^2^ of 5.5 s for the n_H2_ layer, and there was a 5 s pause in each PS layer formation. 

In order to obtain a stable PS substrate, the prepared freshly-etched PS substrates were oxidized by being soaked in H_2_O_2_ (30%) for 24 h. Then the substrates were rinsed with deionized water (DI) thoroughly and dried in air. Oxidization is a necessary step for subsequent functionalization, which results in hydrophilicity for biological application.

### 2.4. Functionalization and Detection

The prepared oxidized PS substrates were dipped into 5% APTES in water/methanol mixture (*v*/*v* = 1:1) for 1 h at RT, then rinsed with deionized water and heated at 100 °C for 10 min to promote cross-linking. After that, the substrates were incubated in 2.5% solution of glutaraldehyde (GA) for 1 h and washed with phosphate buffer saline (PBS, pH: 7.4). The details of functionalization process in this experiment were performed strictly in accordance with our earlier work. In order to immobilize probe DNA, 50 μL probe DNA was dropped onto the oxidized PS substrate at 37 °C for 2 h and washed with PBS three times and dried in air. Then 50 μL of 3 M ethanolamine (EA) was dropped onto the PS substrate at 37 °C for 1 h to prevent non-specific absorption. Fifty microliters of different concentrations of target DNA was dropped onto the PS biosensor at 37 °C for 2 h and then washed with PBS buffer three times to remove the excess target DNA and dried in air. After each step, the reflectance spectra of the PS substrate were measured by a UV-VIS spectrophotometer to verify the success of the PS biosensor.

## 3. Results and Discussions

**Figure 3:**
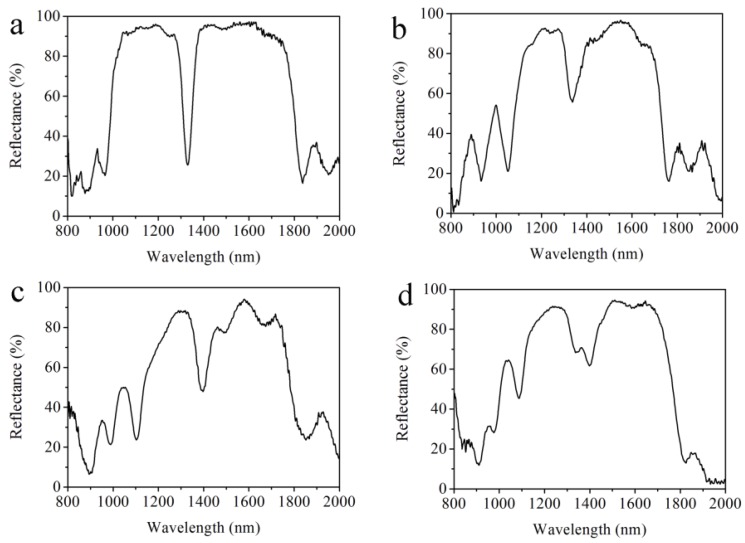
Experimentally-measured reflectance spectra of the double Bragg mirror structure following (**a**) (n_L1_n_H1_)^9^(n_L2_n_H2_)^9^; (**b**) (n_L1_n_H1_)^10^(n_L2_n_H2_)^10^; (**c**) (n_L1_n_H1_)^11^(n_L2_n_H2_)^11^; and (**d**) (n_L1_n_H1_)^12^(n_L2_n_H2_)^12^ sequences.

In the electrochemical etching process, there are longitudinally electrochemical etching and horizontally chemical etching. The anisotropy of the etching rate during the formation of the multilayered PS increases with the time, which breaks the homogeneity of the double Bragg mirror structure. Under this consideration, the number of periods of the double Bragg mirror should be optimized to benefit the quality factor for a large number of repetitions. Such a study plays a vital role in the characterization of the PS double Bragg mirror structure for accurate and correct sensing of biological molecules and is considered in Figure 3.

Figure 3 shows the reflectance spectra of a double Bragg mirror of a (n_L1_n_H1_)^m^(n_L2_n_H2_)^m^ sequence with a different number of periods. From Figure 3a, we can see that the width of the high-reflectivity stop band is about 761 nm and the reflectance is about 95.6% with a sharp resonance peak at 1328 nm in the measured reflectance spectrum of the double Bragg mirrors following the (n_L1_n_H1_)^9^(n_L2_n_H2_)^9^ sequence. The reflectance of resonance peak is about 24% and the full width at half maximum (FWHM) is 42 nm, where the quality factor (*Q* = λ/Δλ) is 31.6. Compared to various other PS-based photonic configuration structures [11,14,16,17,19], the PS double Bragg mirror structure has a wider reflectivity stop band and a deeper resonance dip, which means that such a sensor gives a high distinguishablity. In Figure 3b, the width of the high-reflectivity stop band is about 624 nm and the reflectance is about 96%, with a resonance peak at 1336.0 nm in the measured reflectance spectrum of the double Bragg mirrors following the (n_L1_n_H1_)^10^(n_L2_n_H2_)^10^ sequence. The resonance peak is about 56% and the full width at half maximum (FWHM) is 66 nm. In Figure 3c, the width of the high-reflectivity stop band is about 633.7 nm and the reflectance is about 93.8%, with a resonance peak at 1393.9 nm in the measured reflectance spectrum of the double Bragg mirrors following the (n_L1_n_H1_)^11^(n_L2_n_H2_)^11^ sequence. The reflectance of the resonance peak is about 56% and the full width at half maximum (FWHM) is 63.5 nm. In Figure 3d, the width of the high-reflectivity stop band is about 612.5 nm and the reflectance is about 94.3%, with a resonance peak at 1396.3 nm in the measured reflectance spectrum of the double Bragg mirrors following the (n_L1_n_H1_)^12^(n_L2_n_H2_)^12^ sequence. The resonance peak is about 62.1%, but the optical properties of the defect are obviously destroyed. It can be obviously seen from Figure 3, with the increase of the number of periods, that the resonance peak of the reflectance spectrum moves to a longer wavelength, from 1328.0 nm to 1396.3 nm. Furthermore, the optical properties of the PS double Bragg reflector are gradually destroyed. The best results are obtained when the double Bragg mirrors follow the (n_L1_n_H1_)^9^(n_L2_n_H2_)^9^ sequence, which agrees with the calculated reflectance spectrum and gives a higher Q factor. The difference between the calculated and the measured reflectance spectra comes from the effect of a long residence in the electrolyte. With the etching time increasing, horizontally chemical etching destroys the homogeneity of the PS structure more and the detrimental effects of a depth heterogeneity in the porosity for the first layers, resulting in the destruction of the optical properties of the multilayered PS structure. The optical properties of the double Bragg mirrors are gradually destroyed with the increase of the number of layers. Thus, the proper number of layers and periods are important for the optical properties of the PS double mirror. Furthermore, non-equal surface current density, fabrication tolerance and the loss factor value of a bulk Si will affect the properties of each PS layer in double Bragg mirror. Compared to various other one-dimension PS photonic structures, such a PS double Bragg mirror structure following the (n_L1_n_H1_)^9^(n_L2_n_H2_)^9^ sequence with excellent optical properties is quite easy to prepare and has high sensitivity and specificity.

**Figure 4:**
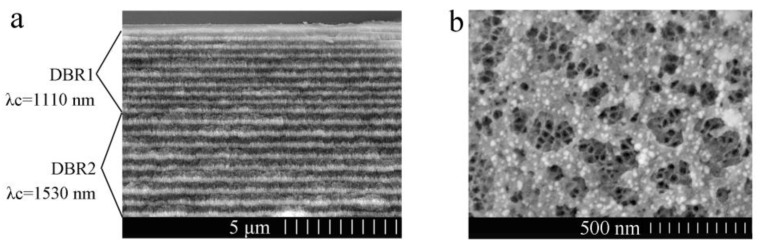
(**a**) Cross-sectional FESEM image of the double Bragg mirrors following the (n_L1_n_H1_)^9^(n_L2_n_H2_)^9^ sequence. (**b**) Top view of the surface.

Figure 4a shows a cross-sectional image of the PS double Bragg mirror structure following the (n_L1_n_H1_)^9^(n_L2_n_H2_)^9^ sequence. The dark color layers are related to the low refractive index (n_L1_ and n_L2_) PS layer with high porosity, and the light gray colored layers are related to the high refractive index (n_H1_ and n_H2_) PS layer with low porosity. The thickness of nL1 or n_H1_ of DBR1 is about 145 nm or 200 nm. The thickness of n_L2_ or n_H2_ of DBR2 is about 196 nm or 280 nm. The thickness of each layer is consistent with the simulation results and there is a strong contrast between each alternate layer, which confirms that a large porosity difference has been achieved. However, there are some slight heterogeneities and variations in the transverse dimension of cross-sectional SEM image, which is mainly related to the nonuniformity of the applied current density distribution. Figure 4b shows a surface image of the PS double Bragg mirrors. The average diameter of the pores for high porosity is approximately from 70 nm to 200 nm and the pore distribution is random. Furthermore, we can see the average diameter of low porosity for the PS layer is about 30 nm through the first PS layer, which means that the biomolecule can infiltrate into the PS double Bragg mirrors well.

**Figure 5:**
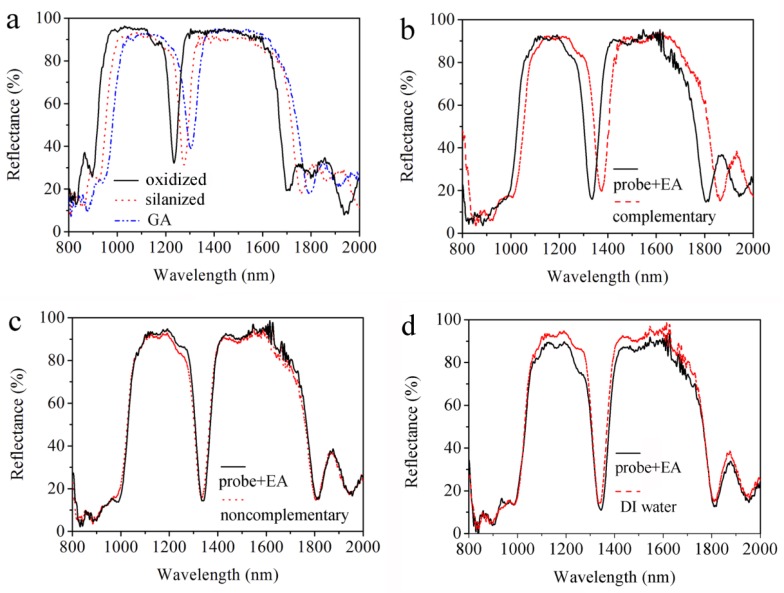
(**a**) Reflectance spectra of double Bragg mirror structure following (n_L1_n_H1_)^9^(n_L2_n_H2_)^9^ sequence after oxidization, after silanization and after glutaraldehyde (GA). (**b**) Resonance shift of the double Bragg mirrors for 10 μM complementary deoxyribonucleic acid (DNA). Negligible resonance shift for (**c**) 10 μM non-complementary DNA and (**d**) Deionized water (DI).

For the reflectance spectra, PS was found to be extremely sensitive to the presence of dielectric substances inside the pores due to the increase of the effective refractive index of a PS layer as the pores are filled, shifting the reflectance spectrum of the layer to longer wavelengths. In this experiment, the reflectance spectrum of the double Bragg mirror demonstrates the success of functionalization, which is performed strictly in accordance with the experimental procedures in our earlier works [11,14,17]. Figure 5a shows that the reflectance peaks for oxidization (oxidized), silanization (silanized) and glutaraldehyde (GA) treatment are 1234.4, 1277.0, and 1304.6 nm, respectively. The redshifts of the reflectance peaks are attributed to the increase of the refractive index for each PS layer. Furthermore, the effective index of PS increases due to the small organic molecules being coupled together well. Thus, the functionalization has been successfully carried out. There is a ca. 10% decrease in reflectance after the GA functionalization in Figure 5a. GA is a colorless, transparent, oily liquid with a two-way coupling agent, and a part of it remains after being reacted with the PS surface rinsed with PBS, which affects the intensity of the refractive index. 

Figure 5b demonstrates the effective DNA detection by the reflectance spectra of the PS double Bragg mirror before and after 10 μM of complementary DNA being attached, where the resonant peak of the double Bragg mirror redshifts from 1334.78 nm to 1379.13 nm due to the increases of the effective refractive index of the PS layer with specific hybridization of probe DNA and complementary DNA. To demonstrate the specificity, controlled experiments were performed by using non-complementary DNA and DI water in Figure 5c,d. The refractive index remained unchanged and almost no shift was detected after non-complementary DNA and DI water being dropped onto two PS biosensors, respectively. This is due to the probe DNA and non-complementary DNA not being bonded in the pores of the PS biosensors and, thus, the refractive index remains unchanged. Figure 5 indicates that the change of the reflectance spectrum is able to select DNA hybridization between complementary DNA and non-complementary DNA.

**Figure 6:**
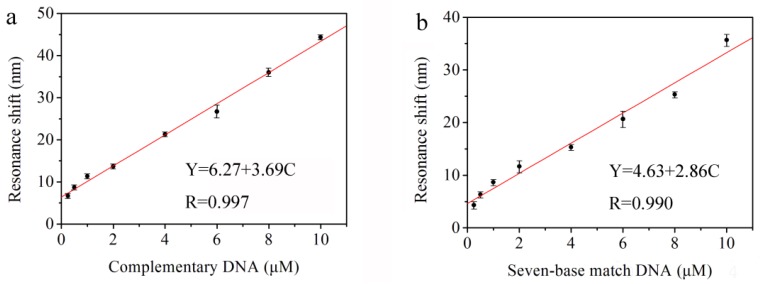
Measured resonance red-shifts of double Bragg mirror structure following the (n_L1_n_H1_)^9^(n_L2_n_H2_)^9^ sequence after being exposed to different concentrations of (**a**) complete complementary DNA and (**b**) partial complementary DNA.

Figure 6a shows the redshifts of the reflectance resonant peak for 18-based complementary DNA with different complete concentrations (i.e., 0.25, 0.5, 1.0, 2.0, 4.0, 6.0, 8.0, and 10.0 μM). The different redshift values are caused by the probe DNA and complementary DNA hybridized in the pores of the PS double Bragg mirror substrate followed by an increase of the refractive index of the PS layer. Furthermore, the average redshift values of the resonance peak corresponding to the concentrations of the complementary DNA are 6.7, 8.7, 11.3, 13.7, 21.3, 26.7, 36.0, and 44.3 nm, respectively. The reproducibility of this DNA sensor has been undertaken in triplicate for three substrates under the same conditions and the reproducibility is good. The linear fitness of the experimental redshift data in Figure 6a provides the sensitivity of the biosensor. The linear equation is Y = 6.27 + 3.69 C, and the correlation is 0.997, where Y represents the redshift value of the resonance peak and C represents the concentration of complementary DNA. The sensitivity of the DNA biosensor is 3.69 nm/μM calculated by the linear fit slope. Therefore, the resolution of the UV-VIS spectrophotometer is 0.1 nm and the lowest detection limit of the biosensor is 0.1/3.69 = 27.1 nM. This method to determine LOD can be found in [14,17,24,25].

In order to verify the effectiveness of biosensor for a real sample. We used DNA with only seven base pairs complementary to the probe DNA as the detection primers and the primers, themselves, are not complementary. The results show that there is still an obvious redshift of the reflectance spectra for the PS double Bragg mirrors as a function of the different concentration of partial complementary DNA (i.e., 0.25, 0.5, 1.0, 2.0, 4.0, 6.0, 8.0, and 10.0 μM). The average redshift values of the resonance peak related to the concentrations of the partial complementary DNA are 4.3, 6.3, 8.7, 11.7, 15.3, 20.7, 25.3, and 35.7 nm, respectively. The linear fitness of the experiment data is given in Figure 6b. The linear equation is Y = 4.63 + 2.86 C, and the correlation is 0.990, where *Y* represents the redshift value of the resonance peak and C represents the concentration of partial complementary DNA. The sensitivity of the DNA biosensor is 2.86 nm/μM from the slope and the resolution of the UV-VIS spectrophotometer is 0.1 nm. Thus, the limit of detection (LOD) of the biosensor is 0.1/2.86 = 35.0 nM. It can be concluded that the PS sensor of this double Bragg mirror structure can detect the complete complementary and partial complementary DNA, which provides the feasibility for the detection of AOB in a real environment. Compared to the complementary DNA in Figure 6a, the redshift value of partial complementary DNA is smaller with lower sensitivity. Furthermore, the DNA hybridization detection limit based on PS double Bragg mirror is of the nM concentration, which is comparable to other reported favorable one-dimension photonic crystal biosensor based on PS, such as the novel PS polybasic symmetrical structure for detecting 23-base pair DNA with a detection limit of 21.3 nM [14], a microcavity biosensor on silicon-on-insulator for detecting 19-base pair DNA with a detection limit of 43.9 nM [17], and a PS membrane waveguide for 24-base pair DNA with a detection limit of 42.0 nM [24]. Considering that the fabricated PS double Bragg mirror structure by electrochemical etching is simple and controllable, this research could be interesting for developing a new optical label-free biosensor based on PS. Furthermore, the broad and high-reflectivity stop band gap with deep and narrow resonance peak in the reflectance spectrum provides great potential for the development of optical applications.

## 4. Conclusions

Through simulations and experiments, a high-sensitivity label-free DNA biosensor based on a PS double Bragg mirror structure for detecting AOB has been shown. The width of the high-reflectivity stop band is about 761 nm with a sharp and deep resonance peak at 1328 nm in the reflectance spectrum center of the double Bragg mirror following the (n_L1_n_H1_)^9^(n_L2_n_H2_)^9^ sequence, which gives a higher sensitivity and distinguishability for sensing performance. There is a significant redshift in the reflectance spectrum when either complete complementary or partial complementary AOB is infiltrated into the pores, which results in the changing of the PS layers’ refractive indices. The detection sensitivity of this biosensor is determined through the investigation of AOB DNA hybridization in the pores, with a detection limit of 27.1 nM. In addition, the lowest detection limit of the biosensor for partial complementary DNA is 35.0 nM, which provides the feasibility and effectiveness for the detection of AOB in a real environment. Easy fabrication, small molecule detection capability, low design cost, and excellent optical properties make the PS double Bragg mirror structure attractive for widespread biosensing applications and provides great potential for the development of optical communication.
